# Methotrexate Treatment Suppresses Monocytes in Nonresponders to Pneumococcal Conjugate Vaccine in Rheumatoid Arthritis Patients

**DOI:** 10.1155/2022/7561661

**Published:** 2022-07-28

**Authors:** Evelina Elmér, Per Nived, Åsa Pettersson, Lillemor Skattum, Thomas Hellmark, Meliha C. Kapetanovic, Åsa C. M. Johansson

**Affiliations:** ^1^Department of Laboratory Medicine, Hematology and Transfusion Medicine, Lund University, Lund, Sweden; ^2^Department of Clinical Immunology and Transfusion Medicine, Region Skåne, Lund, Sweden; ^3^Department of Infectious Diseases, Central Hospital, Kristianstad, Sweden; ^4^Department of Clinical Sciences Lund, Rheumatology, Lund University and Skåne University Hospital, Lund, Sweden; ^5^Department of Clinical Sciences Lund, Nephrology, Lund University and Skåne University Hospital, Lund, Sweden; ^6^Department of Laboratory Medicine, Microbiology, Immunology and Glycobiology, Lund University, Lund, Sweden; ^7^Department of Clinical Genetics and Pathology, Region Skåne, Lund, Sweden

## Abstract

Patients with rheumatoid arthritis (RA) have an increased risk of infections; therefore, immunization against vaccine-preventable diseases is important. Methotrexate (MTX) impairs the antibody response to pneumococcal conjugate vaccine (PCV) in patients with arthritis, and the underlying mechanism is largely unknown. Here, we investigate the potential role of the innate immune system in the faltering antibody response following PCV vaccination in RA patients treated with MTX. Phenotypes of circulating granulocytes and monocytes were analyzed in 11 RA patients treated with MTX, 13 RA patients without disease-modifying antirheumatic drug treatment (0DMARD), and 13 healthy controls (HC). Peripheral blood samples were collected before and 7 days after vaccination. In addition, the MTX group was sampled before initiating treatment. Frequencies of granulocyte and monocyte subsets were determined using flow cytometry. Serotype-specific IgG were quantified using a multiplex bead assay, pre- and 4-6 weeks after vaccination. At baseline, no differences in granulocyte and monocyte frequencies were observed between the groups. Within the MTX group, the frequency of basophils increased during treatment and was higher compared to the HC and 0DMARD groups at the prevaccination time point. MTX patients were categorized into responders and nonresponders according to the antibody response. Before initiation of MTX, there were no differences in granulocyte and monocyte frequencies between the two subgroups. However, following 6-12 weeks of MTX treatment, both the frequency and concentration of monocytes were lower in PCV nonresponders compared to responders, and the difference in monocyte frequency remained after vaccination. In conclusion, the suppressive effect of MTX on monocyte concentration and frequency could act as a biomarker to identify nonresponders to PCV vaccination.

## 1. Introduction

Rheumatoid arthritis (RA) is an autoimmune disease characterized by chronic inflammation of the synovium and erosions in peripheral joints [1]. Several types of immune cells including B cells, T cells, and macrophages have been suggested to contribute to the inflammation in RA. B cells activate T cells and secrete autoantibodies, such as rheumatoid factor (RF) and anticitrullinated protein antibodies (ACPA), and proinflammatory cytokines. T cells promote inflammation by activating fibroblasts and macrophages which release cytokines and chemokines [2]. The clinical presentation is highly variable, and some individuals are negative for autoantibodies (seronegative RA). The disease etiology is complex and involves a combination of genetic susceptibility and environmental factors [3].

Patients with RA, as well as other autoimmune diseases, exhibit an increased risk of infections. The cause is multifactorial and likely a combination of the autoimmune nature of the disease and available pharmacological treatments such as glucocorticoids and different types of disease-modifying antirheumatic drugs (DMARDs) [4, 5]. Therefore, immunization against vaccine-preventable diseases is important in RA [4].

Methotrexate (MTX), the most commonly used DMARD and first-line treatment in RA [2], is known to decrease the humoral response to several vaccines, including seasonal influenza and pneumococcal vaccines in RA patients [6–9]. Methotrexate was initially developed in the oncology field and is a folate antagonist inhibiting DNA and RNA synthesis. However, low-dose treatment with MTX suppresses inflammation in RA by regulating many proinflammatory cell lineages, via mechanisms involving adenosine signaling, inhibition of nuclear factor-*κ*B (NF-*κ*B), inhibition of dihydrofolate reductase-related transmethylation reactions, and generation of reactive oxygen species via nitric oxide synthase [10].

To date, it is not known which mechanism is mainly responsible for the decreased antibody response to vaccination in MTX-treated RA patients. B cell-activating factor (BAFF) promotes B cell activation and differentiation for antibody production. Park et al. have demonstrated that high levels of BAFF negatively impact the response to seasonal influenza vaccine in MTX-treated RA patients [11].

To explore the decreased antibody response to 13-valent pneumococcal conjugate vaccine (PCV13) in MTX-treated RA patients, our group has previously investigated phenotypic changes of circulating B and T cells. Methotrexate treatment reduced the frequency and concentration of Th17 cells and attenuated the activation of plasmablasts and switched memory B cells following PCV vaccination [12].

The innate and adaptive immune systems act in concert to provide an effective immune response to an immunization [13]. Cells of the innate immune system—monocytes, macrophages, and granulocytes—are crucial links to the adaptive immune response via antigen presentation, phagocytosis, and cytokine production.

Monocytes are precursors of tissue macrophages and monocyte-derived dendritic cells and expand during inflammation. They have pattern recognition receptors, which enables them to react to pathogens and produce cytokines. Moreover, monocytes have additional functions, including antigen presentation. Depending on the condition, monocytes can act either proinflammatory via presentation of antigens to T cells and induction of specific T cell subsets or anti-inflammatory via promotion of regulatory T cells and suppression of T cell proliferation. In contrast, some reports suggest that monocytes are poor antigen presenters, only transporting antigen to the lymph nodes [14].

Monocytes can be subdivided into three subsets based on their expression of the lipopolysaccharide (LPS) receptor, CD14, and the Fc*γ*IIIR, CD16. Classical monocytes express high levels of CD14 but no CD16, intermediate monocytes show high levels of CD14 and low CD16, and nonclassical monocytes express low levels of CD14 and high CD16 [15]. The intermediate and nonclassical (CD16^+^) monocytes are considered inflammatory and have been shown to be increased in RA [16, 17]. Further, increased frequency of CD16^+^ monocytes in RA patients has been shown to correlate with active disease, defined as increased number of tender and swollen joints, levels of acute-phase reactants, and titer of rheumatoid factor [16].

Granulocytes are the most abundant leukocytes in blood and the first line of defense against pathogen invasion. Apart from their antimicrobial activity, granulocytes have in recent years been shown to possess other biological functions important in shaping adaptive immunity by interacting with other immune cells and act as antigen-presenting cells under certain conditions [18, 19].

We hypothesized that the innate immune system is of importance for the faltering antibody response to PCV vaccination in RA patients treated with MTX. Using flow cytometry, we monitored circulating granulocyte and monocyte subsets during MTX treatment and after PCV vaccination in RA patients and controls.

## 2. Materials and Methods

### 2.1. Patients and Controls

Adult RA patients, either planned to start methotrexate treatment (MTX group) or without ongoing/planned disease-modifying antirheumatic drug treatment (0DMARD group), at the Department of Rheumatology, Skåne University Hospital, Lund, Sweden, were consecutively included in this study from February 2018 to November 2019. Patients had to fulfil the American College of Rheumatology/European League Against Rheumatism criteria for RA [20].

Patients were not included if they had been treated with DMARDs within 6 months, were treated with prednisolone > 15 mg/day, if they had previously received pneumococcal vaccine, had a history of allergic reaction at previous vaccination, were pregnant, or had an ongoing infection.

Healthy controls (HC) were recruited from the staff at the Department of Rheumatology in Lund and their relatives, between November 2018 and April 2019. There was no age or gender matching between the patients and HC.

The study was approved by the regional ethical review board in Lund, Sweden (permit number 2016/143). Prior to inclusion, all subjects gave written informed consent.

### 2.2. Measurements and Clinical Parameters

At time of inclusion, a rheumatologist performed a physical examination and clinical data regarding date of RA debut, smoking habits, radiographic changes, presence of subcutaneous nodules, treatment, and previous vaccinations were collected using a structured protocol. Disease activity score (DAS28) [21] was used to assess disease activity in RA. Serum levels of rheumatoid factor (RF) and anticitrullinated protein antibodies (ACPA) were analyzed as routine clinical samples at the Department of Clinical Immunology and Transfusion Medicine, Region Skåne, Lund. C-reactive protein (CRP) in plasma, erythrocyte sedimentation rate (ESR), and white blood cell (WBC) count in blood were analyzed as routine clinical samples at the Department of Clinical Chemistry, Region Skåne, Lund. WBC count in blood was also determined with Sysmex XN-350 (Sysmex Europe GmbH).

### 2.3. Vaccination

All participants received a single 0.5 mL dose of 13-valent pneumococcal conjugate vaccine (PCV13, Prevenar13®, Pfizer) administrated as an intramuscular injection in the deltoid muscle. Prevenar13® includes capsular polysaccharide of 13 serotypes of *Streptococcus pneumoniae* (1, 3, 4, 5, 6A, 6B, 7F, 9V, 14, 19A, 19F, 18C and 23F) conjugated to diphtheria toxin known as CRM197, and aluminium phosphate as adjuvant.

Patients in the 0DMARD group and HC were vaccinated at time of inclusion. Patients in the MTX group were vaccinated 6-12 weeks after initiating MTX treatment, when they had been on unaltered MTX dose for at least four weeks. At time of vaccination, a physical examination was performed, and data on disease activity was collected.

### 2.4. Pneumococcal Serotype-Specific Antibodies

Serum samples were collected from most subjects (10 HC, 8 0DMARD and 9 MTX patients) immediately before administration of PCV13 and 4-6 weeks later. Sera were frozen at -80°C and later analyzed at the Department of Clinical Immunology and Transfusion Medicine, Region Skåne, Lund. Pneumococcal serotype-specific IgG concentrations were measured for 11 capsular serotypes (1, 3, 4, 5, 6B, 7F, 9V, 18C, 19A, 19F and 23F) included in PCV13, using an in-house multiplex fluorescent microsphere immunoassay (MFMI, Luminex) based on the procedure described by Lal et al. [22], with some modifications. Antibody response ratio (ARR, i.e., the ratio of post- to prevaccination serotype-specific IgG concentration) was calculated [7], and positive antibody response was defined as ARR ≥ 2, in > 50% of serotypes (at least 6 of 11 serotypes) [12]. The sum of the absolute change (*μ*g/mL) in pneumococcal serotype-specific IgG concentrations, for the 11 capsular serotypes included in PCV13, pre- to postvaccination, was calculated and denoted composite antibody response.

### 2.5. Phenotypic Characterization of Granulocytes and Monocytes

In the MTX group, peripheral blood samples were collected at inclusion (before initiating MTX treatment), at vaccination (after 6-12 weeks on MTX), and 6-12 days after vaccination (with the majority sampled 6-7 days postvaccination). In the 0DMARD group and HC, blood samples were collected at vaccination (i.e., at inclusion) and 6-7 days after vaccination. Two patients in the 0DMARD group were not included in the analyses after vaccination due to technical problems.

Venous blood was obtained in heparin tubes (BD Vacutainer ref 369622) and stored at room temperature and protected from light until analyzed (within 24 h). The expression of selected surface markers on monocytes, lymphocytes, and granulocytes was analyzed using flow cytometry. Briefly, red blood cells were lysed using 0.84% ammonium chloride. The leukocytes were washed with phosphate-buffered saline (PBS) and resuspended in PBS with 0.5% bovine serum albumin. An antibody cocktail of the following monoclonal fluorescent-labeled antibodies was added to the suspension of leukocytes: CD14 PerCP Cy5.5 (M5E2), CD16 APC-H7 (3G8), CD193 V510 (5E8), CD45 V450 (2D1), HLA-DR PE Cy7 (L243) from BD Biosciences (San Jose, CA, USA), CD80 FITC (BB1) from Santa Cruz Biotechnology (Dallas, Texas, USA), Siglec-8 PE (7C9), CD66b Alexa700 (G10F5), CD11b BV785 (ICRF44), CD62L BV650 (DREG-56), CD69 PE/Dazzle 594 (FN50) from BioLegend (San Diego, CA, USA), CD184 APC (12G5) from eBioscience/Thermo Fisher Scientific (Waltham, MA, USA). Acquisition was performed on a FACSAria Fusion flow cytometer with the accompanying FACSDiva software (Becton Dickinson, Franklin Lakes, NJ, USA). At least 50.000 granulocytes were acquired based on forward and side scatter properties. Data were analyzed using Kaluza Analysis Software version 2.1 (Beckman Coulter, Brea, CA, USA).

Analysis using this panel allowed identification of lymphocytes, basophils, neutrophils, eosinophils, monocytes and subsets. Gating was performed in a blinded manner. Details of the gating strategy are shown in Figure S1. Doublet cells were excluded by plotting forward scatter height against forward scatter area, and CD45^+^ single cells were divided into monocytes, lymphocytes, and granulocytes based on forward and side scatter properties. The basophils in the dim CD45/low side scatter were included in the peripheral blood mononuclear cell (PBMC) gate. PBMCs were plotted with CD45 and CD193 and basophils were gated (dim CD45/high CD193). Basophils were separated from plasmacytoid dendritic cells (pDCs) and other HLA-DR^+^ cells by the absence of HLA-DR expression. CD14-negative polymorphonuclear leukocytes (PMNs) were plotted with Siglec-8 and CD193 for identification of neutrophils (Siglec-8 and CD193 negative) and eosinophils (Siglec-8 and CD193 high). Monocytes were subdivided by the expression of CD14 and CD16 into classical (CD14^++^CD16^−^), intermediate (CD14^++^CD16^+^), and nonclassical (CD14^+^CD16^++^) monocytes. The monocyte surface expression of CD11b, CD62L, CD69, CD80, and HLA-DR was measured by median fluorescence intensity (MdFI).

Absolute numbers were based on the cell population proportion of CD45^+^ cells combined with the WBC count. All values are given as percentage of CD45^+^ cells if not otherwise specified. The main focus was to investigate granulocyte and monocyte frequencies, but lymphocyte frequency was also analyzed (detailed lymphocyte data published in Nived et al.) [12].

### 2.6. Statistical Analysis

Statistical analyses were performed with GraphPad Prism 9.3.1 software (GraphPad Software, San Diego, CA, USA). Mann–Whitney *U* test was used for two-group comparisons and Kruskal-Wallis with Dunn's multiple comparisons test for three or more groups. Paired data were analyzed using Wilcoxon matched-pairs signed rank test.

Statistical analysis was only performed for the parameters considered relevant for answering one or more of the hypotheses of the study. Samples with pneumococcal serotype-specific IgG concentrations below the lower limit of detection (for serotype 9V, *n* = 2, one 0DMARD patient and one HC, both before vaccination) were set to 0. Subgroups of *n* < 4 were not included in the statistical analyses. Values are expressed as median with interquartile range (IQR) unless otherwise specified. Results were considered statistically significant at *p* < 0.05.

## 3. Results

### 3.1. Characteristics of Study Participants

11 RA patients scheduled to start treatment with MTX (MTX group), 13 RA patients without ongoing or planned DMARD treatment (0DMARD group), and 13 healthy controls (HC) were enrolled. Patients in the 0DMARD group and HC were included at time of vaccination, whereas MTX patients were included when initiating MTX treatment. Demographics and characteristics of study participants are described in Table 1. The healthy controls were younger than the RA patients (*p* = 0.008). There were more females than males in all three groups, with the highest female to male ratio in the MTX group and the lowest in the HC group. Compared to the 0MDARD group, the majority of patients in the MTX group was older at RA debut (*p* < 0.05), had shorter disease duration (*p* = 0.02), higher disease activity score (*p* = 0.008), and higher ESR (*p* = 0.04) at inclusion.

### 3.2. MTX Treatment Attenuates Antibody Response following Pneumococcal Vaccination

To evaluate vaccine response, pneumococcal antibody levels were measured in patients and controls. Pneumococcal serotype-specific IgG concentrations of 11 serotypes included in 13-valent pneumococcal conjugate vaccine (PCV13) were analyzed, right before and 4–6 weeks after vaccination. A positive antibody response (≥ twofold increase in ≥ 6 serotypes pre- to postvaccination) was seen in 90% of HC, 87.5% of the 0DMARD group, and 56% of the MTX group. Number of serotypes with at least twofold increase in antibody level was only significantly different between HC and MTX groups (9.00, 8.25-11.0 and 6.00, 1.00-7.50, respectively, *p* = 0.02). Serotype-specific antibody responses are shown in Table S1 and Figure S2.

Antibody response ratio (ARR, the ratio of post- to prevaccination antibody levels) was lower in the MTX group for five serotypes compared to HC (Figure S3a). Furthermore, changes in antibody titers pre- to postvaccination, in absolute values, were lower in the MTX group for three serotypes compared to HC and for one serotype compared to 0MDARD (Figure S3b).

**Figure 1:**
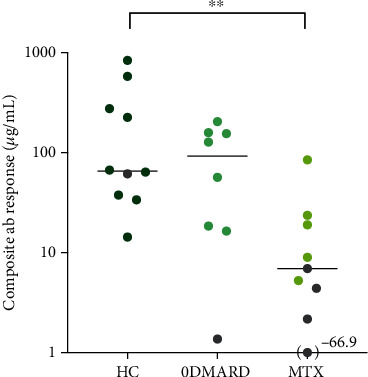
Composite antibody response in healthy controls, RA 0DMARD, and RA MTX groups, after immunization with 13-valent pneumococcal conjugate vaccine (PCV13). Composite antibody response represents the sum of change in pneumococcal serotype-specific IgG concentrations (*μ*g/mL), for 11 capsular serotypes included in PCV13, pre- to postvaccination. Nonresponders are depicted in grey and the remaining are responders (defined as ≥ twofold increase in antibody titers in ≥ 6 serotypes pre- to postvaccination). Kruskal-Wallis with Dunn's multiple comparisons test was used to calculate level of significance. Data are presented with medians. ab: antibody; HC: healthy control; 0DMARD: without disease-modifying antirheumatic drug treatment; MTX: methotrexate; RA: rheumatoid arthritis. Antibody titers were measured in 10 HC, 8 0DMARD and 9 MTX patients.

The composite antibody response, i.e., the sum of change in pneumococcal serotype-specific IgG concentrations (*μ*g/mL), for the 11 capsular serotypes included in PCV13, pre- to postvaccination, is depicted in Figure 1. The composite antibody response was lower in the MTX group compared to HC (*p* = 0.006).

### 3.3. No Differences in Cell Frequencies at Baseline

Patients and HC were sampled at inclusion, i.e., prior to initiation of MTX medication (MTX group) or vaccination (0DMARD and HC). There were no differences in percentages and concentrations of total monocytes and granulocytes, their subsets, or total lymphocytes, between MTX, 0DMARD, and HC groups at baseline (Table 2 and data not shown). If combining the MTX and 0DMARD patients to one RA group (patients were not treated with any DMARD at baseline), the percentage of CD14^+^CD16^++^ monocytes (of monocytes) was lower in RA patients compared to HC (*p* = 0.03, Table S2).

### 3.4. Higher Frequency of Basophils during MTX Treatment

**Figure 2:**
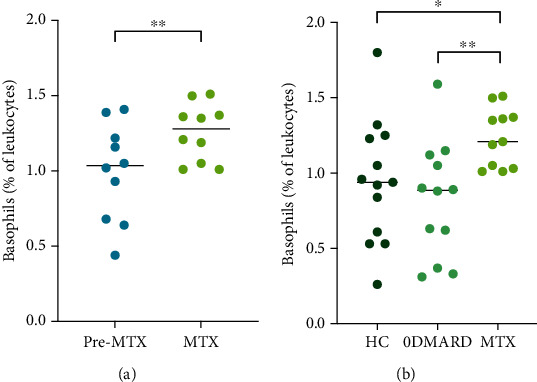
Percentage of basophils (of leukocytes) in peripheral blood from (a) patients with rheumatoid arthritis before methotrexate treatment and after 6-12 weeks on methotrexate, (b) healthy controls (HC), patients without disease-modifying antirheumatic drug treatment (0DMARD group), and patients on MTX treatment (MTX), before administration of 13-valent pneumococcal conjugate vaccine. Wilcoxon matched-pairs signed rank test and Mann–Whitney *U* test were used to calculate level of significance. Data are presented with medians.

To investigate the impact of MTX on monocyte and granulocyte frequencies, these were analyzed before initiation of MTX treatment and at vaccination, i.e., when patients had been on unaltered MTX dose for at least four weeks (6-12 weeks of total treatment duration). The percentage of basophils increased during MTX treatment (*p* = 0.01, Figure 2(a)), and there was a tendency of higher concentration of basophils (0.067 × 10^9^/L, 0.055-0.087 and 0.086 × 10^9^/L, 0.057-0.11, *p* = 0.09). Moreover, the percentage of basophils was higher in the MTX group compared to HC and 0DMARD before vaccination (*p* = 0.02 and *p* = 0.006, respectively, Figure 2(b)). No other significant changes in cell frequencies were induced by MTX treatment.

The percentage of eosinophils was higher in the MTX group compared to the 0DMARD group (3.57%, 2.54-5.84 and 1.71%, 0.938-4.69, respectively, *p* = 0.04), and there was a tendency of higher percentage of eosinophils compared to HC (3.00%, 1.60-3.29, *p* = 0.08).

### 3.5. Frequencies of Monocytes, Granulocytes, and Lymphocytes in relation to Methotrexate Treatment and Vaccination with Pneumococcal Conjugate Vaccine

To investigate the underlying mechanisms by which MTX exerts its effect on antibody response, frequencies of circulating monocytes, granulocytes, and lymphocytes were analyzed in RA patients and healthy controls, 6-7 days after administration of PCV13.

Matched-paired analysis of patients with MTX treatment, before and after vaccination, displayed no differences in monocyte, granulocyte, or lymphocyte frequencies or concentrations, except for a lower lymphocyte concentration after vaccination (*p* = 0.02, Table S3), while patients with no DMARD had higher frequency of basophils after vaccination (*p* = 0.002, Table S4). Further, healthy controls showed higher frequency of eosinophils (*p* = 0.01, Table S5).

Apart from a tendency of higher percentage of eosinophils in the MTX patients compared to 0DMARD group (*p* = 0.05, Table 3), there were no conclusive differences between the MTX, 0DMARD, and HC groups, after administration of PCV.

### 3.6. Lower Percentage of Monocytes in Nonresponders to Pneumococcal Conjugate Vaccine in RA Patients with MTX Treatment

As previously demonstrated and confirmed in the present study, MTX impairs the antibody response to pneumococcal conjugate vaccine (PCV) [6–9]. To explore underlying pathophysiological mechanisms, the cellular phenotype and laboratory features between MTX-treated vaccine responders and nonresponders were compared.

Antibody titers pre- and postvaccination were available for nine out of eleven patients with MTX treatment. Five patients displayed a positive antibody-response according to the definition (≥ twofold increase in antibody titers in ≥ 6 serotypes pre- to postvaccination) and four were nonresponders.

There were no statistical differences between the groups regarding age, disease duration, disease activity (DAS28), or MTX dose. Before initiating MTX treatment, there were tendencies to higher CRP and ESR in nonresponders compared to responders (*p* = 0.1 and *p* = 0.06, respectively, Table S6). At the time of vaccination, i.e., when they had been on unaltered MTX dose for at least four weeks, ESR was significantly higher in nonresponders (*p* = 0.03, Table S6). Demographics and clinical data of MTX-treated responders and nonresponders are presented in Table S6.

**Figure 3:**
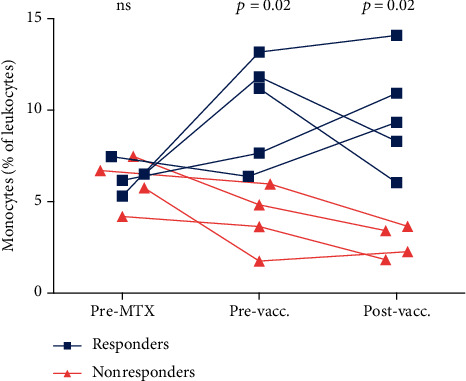
Comparison of monocytes (% of leukocytes), before initiation of methotrexate (MTX) treatment (pre-MTX), with MTX treatment for 6-12 weeks and before vaccination (pre-vacc.), and 6-7 days after administration of 13-valent pneumococcal conjugate vaccine (post-vacc.), in peripheral blood from rheumatoid arthritis patients, sorted in responders and nonresponders to the vaccine. Positive antibody response was defined as an antibody response ratio (ARR, i.e., the ratio of post- to prevaccination antibody levels) ≥ 2, in > 50% of serotypes. Flow cytometry data was not available for one patient (responder) pre-MTX.

We continued by investigating cell frequencies at the three different time points, pre-MTX, pre- and postvaccination. Before initiation of MTX, there were no differences observed in granulocyte, monocyte, or lymphocyte frequencies or concentrations between responders and nonresponders, except for a tendency of higher granulocyte concentration in nonresponders (Figure 3 and data not shown). After 6-12 weeks of MTX treatment (prevaccination), the percentage and concentration of monocytes were lower in nonresponders (*p* = 0.02 and *p* = 0.03, respectively, Figure 3 and Table 4 and data not shown). The lower percentage of monocytes in nonresponders remained after vaccination (*p* = 0.02, Figure 3, Table S7). In addition, there was a tendency of higher frequency of granulocytes in nonresponders postvaccination (*p* = 0.1, Table S7).

The monocyte population was further divided into classical, intermediate, and nonclassical monocytes. The subsets did not differ between responders and nonresponders, but there was a tendency of lower concentration of classical (CD14^++^CD16^−^) monocytes in nonresponders before vaccination (0.49 × 10^9^/L, 0.42-0.64 and 0.28 × 10^9^/L, 0.072-0.40, *p* = 0.06). After vaccination, there were weak tendencies of lower percentage of classical monocytes and higher percentage of nonclassical (CD14^+^CD16^++^) monocytes in nonresponders (*p* = 0.1 for both comparisons, Table S7).

The percentage and concentration of eosinophils were higher in nonresponders after 6-12 weeks of MTX treatment (*p* = 0.02 and *p* = 0.03, respectively, Table 4 and data not shown), but these differences were not present after vaccination (Table S7).

As a result of the difference in monocyte frequency between responders and nonresponders that developed during MTX treatment, we wanted to further explore the monocyte population in respect to level of activation. Surface expression of CD11b (leukocyte adhesion and migration), CD62L (L-selectin, adhesion to activated endothelial cells), CD69 (upregulated upon activation), CD80 (B7-1), and HLA-DR (antigen-presenting capacity) on circulating monocytes was analyzed in responders and nonresponders, pre-MTX, pre- and postvaccination. There were no statistical differences in expression of these activation markers on monocytes and subsets between responders and nonresponders at the three different time points (Table S8).

## 4. Discussion

Autoimmune inflammatory rheumatic diseases are accompanied by an increased risk of infections; therefore, immunization against vaccine-preventable diseases is important. In this study, we found that MTX-treated nonresponders to PCV display lower frequency and concentration of monocytes compared to responders.

At inclusion, there were no differences in cell percentages or concentrations between MTX, 0DMARD, and HC groups. However, if combining the patient groups, the percentage of nonclassical (CD14^+^CD16^++^) monocytes was lower in RA patients compared to HC. This is partly in contrast with previous studies that have demonstrated both higher and unchanged monocytes and subsets in untreated RA patients as compared to HC [23–25]. The different results reported in untreated patients could possibly be related to disease activity and duration. In Chara et al., patients not responding to MTX showed higher absolute number of circulating monocytes, before starting and throughout treatment. Responders to MTX showed normal numbers of monocytes, and their subsets cells, over the study period [24].

Here, we demonstrate that 6-12 weeks of MTX treatment induced only minor alterations in circulating granulocytes and monocytes. The frequency of basophils increased in the MTX group, as well as in relation to the 0DMARD and HC groups, and the frequency of eosinophils was higher in the MTX group compared to the 0DMARD group, at the prevaccination time point. The relevance of these findings for the decreased immune responsiveness in MTX-treated patients is unclear. Basophils have been implicated in RA pathogenesis by affecting the Th1/Th2 balance [26], and decreased basophil/lymphocyte ratio and increased eosinophil/lymphocyte ratio have been demonstrated in inflammatory diseases including RA [27]. However, in a prospective observational study, a majority of RA patients had a secondary cause of eosinophilia, and DMARDs did not influence the eosinophil blood count [28].

Using vaccination with pneumococcal vaccine (PCV) as a model for antigen challenge, our group has previously investigated phenotypic changes of circulating B and T cells in RA patients. The hypothesis was that MTX treatment could exert a negative effect on the formation of germinal center follicular T helper cells, resulting in a decreased number of circulating memory follicular T helper cells after vaccination. We demonstrated that MTX treatment reduced the frequency and concentration of Th17 cells and attenuated expansion of plasmablasts and switched memory B cells following PCV vaccination [12].

In the present study, vaccination of MTX-treated patients with PCV induced no significant alterations in monocyte and granulocyte frequencies or concentrations. Moreover, there were no differences between the MTX, 0DMARD, and HC groups, after administration of PCV. However, patients and controls were sampled 6-7 days following vaccination. An earlier sampling time point could possibly have generated more information on changes in cell frequencies induced by the local inflammation and vaccine response. Diks et al. observed increased levels of neutrophils and monocytes, with expansion of intermediate and nonclassical monocytes, up to 5 days after pertussis booster vaccination [29]. Furthermore, an increase in frequency and activation of dendritic cells and monocyte subsets has been observed 1 and 3 days after Ebola vaccination [30].

Two different but partially overlapping meta-analyses based on twelve and nine studies, respectively, concluded that MTX exposure diminishes the antibody response to pneumococcal vaccination. For influenza vaccination, the supporting data were more contradictory [8, 9]. Park et al. demonstrated that holding MTX for 4 weeks (2 weeks before and 2 weeks after vaccination or 4 weeks postvaccination) increased the response to quadrivalent seasonal influenza vaccination [31, 32]. This data further supports the direct role of MTX in decreased immune responsiveness in RA.

Here, to investigate how MTX impairs the antibody response to PCV, patients were divided into responders and nonresponders to PCV. There is no generally accepted definition of what constitutes an adequate serotype-specific antibody response following immunization with PCV [33]. In this study, we defined responders to PCV as those with ≥ twofold increase in antibody titers in ≥ 6 serotypes pre- to postvaccination, in line with previous studies [7, 12].

The main finding was a difference in monocyte frequency between responders and nonresponders that developed following MTX treatment. The frequency was lower in nonresponders compared to responders, and this disparity was sustained after PCV administration.

MTX displays a large number of effects of which several are postulated to be anti-inflammatory, e.g., reversal of T cell cycle checkpoint abnormalities and resistance to apoptosis, decreased activity of NF-*κ*B in T cells, and activation of adenosine receptors in fibroblast-like synoviocytes [10]. In monocyte cell lines, MTX has displayed proinflammatory properties with induction of apoptosis and increased production of IL-1, TNF, and IL-6 [34].

We noticed a lower frequency of classical monocytes and a higher frequency of inflammatory monocytes in nonresponders following MTX treatment; however, these changes were not significant.

Several studies indicate an inverse correlation between frequency of inflammatory monocytes and antibody response, possibly via a defect of T cell help to B cells [35]. Further, Mitchell et al. have shown in murine models and in vitro that interrupting inflammatory monocyte recruitment to lymph nodes leads to enhanced cellular and humoral immune responses to vaccination [36].

Moreover, before MTX treatment, there were tendencies of higher C-reactive protein and erythrocyte sedimentation rate (ESR) in nonresponders compared to responders, and following MTX treatment (at the time of vaccination), ESR was higher in nonresponders. Possibly, the higher level of inflammation could contribute to the decreased antibody response in nonresponders. In support of this, Nakaya et al. have in an extensive study of gene signatures related to immunogenicity of influenza vaccination showed that baseline genetic signatures of monocyte inflammation were negatively correlated with antibody responses at 1 month. They reason that inflammation prevaccination might be unfavorable to the vaccine-induced antibody response [37].

Limitations of the present study are the small sample size and the nonrandomized design. Further, the study groups were not age- or gender-matched, and a limited number of absolute white blood cell counts were available. The healthy controls were younger than the RA patients which might contribute to differences in antibody responses to vaccination, but did not affect the main conclusions within the MTX group or comparisons between the patient groups. In addition, the sampling time following vaccination was not optimal to investigate the innate immune response [29, 30]. Another aspect of the overall aim of the study is the method used to identify responders to the vaccination. Here, we used serotype-specific pneumococcal antibody response ratio (post- to prevaccination) to determine vaccine responsiveness. Analysis of the T cell response to the vaccine (and/or its constituents), as well as opsonophagocytosis, would have added information complementing the serotype-specific antibody response [38].

Monocytes, macrophages, and granulocytes are important to the innate response to vaccine antigens and adjuvant, and are necessary to provide an effective adaptive immune response [13, 29, 39]. Here, we show that MTX exerts an effect on the innate immune system by suppressing monocytes in future nonresponders to PCV vaccination. If reproduced in a larger cohort, the next step would be to explore the underlying mechanisms reflected in the disparity of monocyte frequencies between responders and nonresponders to PCV. For example, knowledge about MTX-induced tissue alterations of cell populations of the innate and adaptive immune system could contribute to the understanding of changes found in circulating immune cells. This could potentially be explored in animal experiments. In summary, monocyte frequency in peripheral blood could have the potential to act as a biomarker to identify future nonresponders to pneumococcal vaccination in MTX-treated RA patients.

## 5. Conclusions

In this limited cohort, we demonstrate a possible role for the innate immune system in the blunted vaccine response of MTX-treated RA patients. The suppressive effect of MTX on monocyte concentration and frequency could act as a biomarker to identify nonresponders to PCV vaccination.

## Figures and Tables

**Table 1:** Demographic profile and characteristics of study participants.

	HC (*n* = 13)	RA 0DMARD (*n* = 13)	RA MTX (*n* = 11)
Age (years), median (IQR)	41.1 (35.9-56.5)	61.2 (43.1-68.0)	62.9 (58.3-66.7)
Female/male, *n* (%)	8/5 (61.5/38.5)	9/4 (69/31)	10/1 (91/9)
Age at debut of RA (years), median (IQR)	NA	41.3 (31.0-58.7)	59.4 (51.4-65.3)
Disease duration (years), median (IQR)	NA	5.5 (0.3-16.6)	0.3 (0.2-0.6)
Current smoker, *n* (%)	0 (0)	4 (31)	2 (18)
Ex-smoker, *n* (%)	3 (23)	2 (15)	5 (45)
Never smoker, *n* (%)	10 (77)	7 (54)	4 (36)
Radiographic changes, yes/no/no data, *n*^a^	NA	6/6/1	4/5/2
Presence of subcutaneous nodules, *n* (%)	NA	3 (23)	0 (0)
RF positive, *n* (%)	—	10 (77)	11 (100)
ACPA positive, *n* (%)	—	11 (85)	5 (45)
DAS28 at MTX start, median (IQR)^b^	NA	NA	5.6 (5.1-6.6)^e^
DAS28 at vaccination, median (IQR)^b^	NA	4.6 (3.1-5.2)	4.6 (3.6-5.6)
CRP at MTX start (mg/L), median (IQR)^c^	NA	NA	8.8 (2.6-32.0)
CRP at vaccination (mg/L), median (IQR)^c^	0.7 (0.6-1.2)	3.5 (1.6-7.0)	2.8 (1.6-8.9)
ESR at MTX start (mm), median (IQR)^d^	NA	NA	51 (28-67)
ESR at vaccination (mm), median (IQR)^d^	6 (3-13)	25 (16-43)	35 (12-45)
Prednisolone at vaccination, *n* (%)	0 (0)	2 (15)	5 (45)
-Dose in treated (mg/day), median (IQR)	NA	3.75 (2.5-5)	5 (2.5-10)
Methotrexate at vaccination (mg/week), median (IQR)	0 (0)	0 (0)	20 (15-25)

HC: healthy controls; RA: rheumatoid arthritis; 0DMARD: without disease-modifying antirheumatic drug treatment; MTX: methotrexate; IQR: interquartile range; NA: not applicable; RF: rheumatoid factor; ACPA: anticitrullinated protein antibody; DAS28: Disease Activity Score 28 joints examined; CRP: (P-CRP) C-reactive protein; ESR: (B-ESR) erythrocyte sedimentation rate. ^a^Fulfil criteria 7 in 1987 Rheumatoid Arthritis Classification. ^b^On a scale 0-10 DAS28 > 5.1 high, 5.1-3.2 moderate, < 3.2-2.6 low disease activity, and < 2.6 remission. ^c^Reference range < 3.0 mg/L. ^d^Reference range < 30 mm (female), < 20 mm (male). ^e^*n* = 10.

**Table 2:** Frequencies of circulating leukocytes in RA patients and HC, at baseline.

Phenotype (% of leukocytes unless otherwise specified)	Healthy controls (*n* = 13)	RA 0DMARD (*n* = 12)	RA MTX (*n* = 10)
Monocytes	5.62 (4.82-7.58)	5.88 (5.04-8.56)	6.34 (5.03-7.47)
CD14^++^CD16^−^ (% of monocytes)	84.5 (79.3-87.8)	86.2 (81.9-91.0)	86.6 (83.7-90.9)
CD14^++^CD16^+^ (% of monocytes)	2.62 (2.04-4.14)	4.77 (2.24-5.55)	4.12 (1.78-4.70)
CD14^+^CD16^++^ (% of monocytes)	13.3 (9.11-16.0)	9.21 (5.41-12.6)	8.63 (5.58-12.9)
Granulocytes	60.1 (43.5-69.6)	66.0 (57.4-82.0)	66.3 (58.3-79.7)
Basophils	0.940 (0.570-1.24)	0.885 (0.433-1.10)	1.04 (0.670-1.26)
Eosinophils	3.00 (1.60-3.29)	1.71 (0.938-4.69)	2.76 (2.13-5.32)
Neutrophils	57.9 (39.2-66.1)	59.4 (55.7-79.0)	60.8 (53.1-76.2)
Lymphocytes	24.8 (20.9-42.0)	20.1 (9.00-32.4)	19.2 (11.6-27.1)

Frequencies of circulating monocytes, granulocytes, and lymphocytes analyzed in patients with RA (in the MTX group before scheduled MTX treatment) and healthy controls, before administration of PCV13, using flow cytometry. Kruskal-Wallis test and Dunn's multiple comparisons test were used to calculate level of significance. There were no statistical differences in frequencies between the three groups. Data are presented with medians and interquartile ranges. RA: rheumatoid arthritis; HC: healthy control, 0DMARD: without disease-modifying antirheumatic drug treatment; MTX: methotrexate.

**Table 3:** Frequencies of circulating leukocytes in RA patients and HC, after vaccination with PCV13.

Phenotype (% of leukocytes unless otherwise specified)	HC (*n* = 12)	0DMARD (*n* = 11)	MTX (*n* = 11)
Monocytes	6.21 (4.59-8.26)	7.19 (5.58-10.5)	7.86 (3.42-10.9)
CD14^++^CD16^−^ (% of monocytes)	82.9 (78.3-86.2)	88.3 (85.5-90.9)	86.5 (80.1-90.1)
CD14^++^CD16^+^ (% of monocytes)	2.93 (2.34-3.42)	2.12 (1.08-4.47)	3.26 (1.39-4.99)
CD14^+^CD16^++^ (% of monocytes)	14.8 (10.8-18.9)	7.99 (6.48-10.9)	10.4 (6.63-13.9)
Granulocytes	58.2 (46.0-73.3)	73.2 (53.6-78.2)	71.0 (60.3-81.0)
Basophils	0.870 (0.595-1.26)	0.960 (0.890-1.38)	1.17 (1.11-1.49)
Eosinophils	2.95 (1.91-4.68)	1.90 (1.66-2.59)	5.73 (2.32-8.17)
Neutrophils	54.0 (41.3-69.5)	70.4 (47.9-75.1)	61.7 (55.8-73.5)
Lymphocytes	30.9 (15.9-37.6)	15.2 (10.4-28.4)	15.4 (9.77-29.0)

Frequencies of circulating monocytes, granulocytes, and lymphocytes analyzed in RA patients on MTX treatment for 6-12 weeks, patients in the 0DMARD group and HC, 6-7 days after administration of PCV13, using flow cytometry. Kruskal-Wallis test and Dunn's multiple comparisons test were used to calculate level of significance. Data are presented with medians and interquartile ranges. RA: rheumatoid arthritis; HC: healthy control; PCV13: 13-valent pneumococcal conjugate vaccine; 0DMARD: without disease-modifying antirheumatic drug treatment; MTX: methotrexate.

**Table 4:** Frequencies of circulating leukocytes in RA patients with MTX treatment, before vaccination with PCV13, sorted in responders and nonresponders.

Phenotype (% of leukocytes unless otherwise specified)	MTX pre-PCV13 Responders (*n* = 5)	MTX pre-PCV13 Nonresponders (*n* = 4)	*p* value
Monocytes	11.2 (7.02-12.5)	4.24 (2.23-5.69)	0.02
CD14^++^CD16^−^ (% of monocytes)	86.9 (83.2-92.0)	68.2 (43.6-90.4)	ns
CD14^++^CD16^+^ (% of monocytes)	3.35 (1.69-6.08)	6.69 (1.10-15.9)	ns
CD14^+^CD16^++^ (% of monocytes)	9.68 (6.36-10.8)	25.1 (6.52-42.5)	ns
Granulocytes	62.2 (58.0-65.0)	70.4 (51.9-77.6)	ns
Basophils	1.19 (1.04-1.51)	1.36 (1.10-1.37)	ns
Eosinophils	2.70 (2.58-3.87)	5.92 (5.03-8.64)	0.02
Neutrophils	58.3 (54.1-59.8)	63.8 (45.0-67.6)	ns
Lymphocytes	24.0 (18.9-27.5)	22.4 (14.0-39.1)	ns

Frequencies of circulating monocytes, granulocytes, and lymphocytes analyzed in RA patients on MTX, immediately before administration of PCV13, using flow cytometry. Positive antibody response was defined as an antibody response ratio (ARR, i.e., the ratio of post- to prevaccination antibody levels) ≥ 2, in > 50% of serotypes. Mann–Whitney *U* test was used to calculate level of significance. Data are presented with medians and interquartile ranges. RA: rheumatoid arthritis; MTX: methotrexate; PCV13: 13-valent pneumococcal conjugate vaccine; ns: not significant. Antibody titers were available in 9 of 11 patients on MTX.

## Data Availability

Raw data files from flow cytometry datasets used in the current study are available from the corresponding author on reasonable request.

## Supplementary Materials

Supplementary Materials Figure S1: gating strategy used to identify lymphocytes, basophils, neutrophils, eosinophils, and monocytes. Figure S2: proportions of individuals with serotype-specific pneumococcal ARR ≥ 2 in healthy controls, RA 0DMARD, and RA MTX groups, after immunization with PCV13. Figure S3: serotype-specific pneumococcal antibody response in healthy controls, RA 0DMARD, and RA MTX groups, after immunization with PCV13. Table S1: distribution of antibody titers pre- and postvaccination of PCV13 in healthy controls, RA 0DMARD, and RA MTX groups. Table S2: frequencies of circulating leukocytes in RA patients, prior to contingent MTX treatment, and HC, before vaccination with PCV13. Table S3: frequencies of circulating leukocytes in RA patients with MTX treatment, before and after vaccination with PCV13. Table S4: frequencies of circulating leukocytes in RA patients without DMARD, before and after vaccination with PCV13. Table S5: frequencies of circulating leukocytes in healthy controls, before and after vaccination with PCV13. Table S6: demographic profile and characteristics of RA patients with MTX treatment, sorted in responders and nonresponders to PCV13. Table S7: frequencies of circulating leukocytes in RA patients with MTX treatment, after vaccination with PCV13, sorted in responders and nonresponders to PCV13. Table S8: expression of activation markers on circulating monocytes in MTX-treated RA patients sorted in responders and nonresponders to PCV13, pre-MTX, pre- and postvaccination.
