# Supplementary material for: Methotrexate Treatment Suppresses Monocytes in Nonresponders to Pneumococcal Conjugate Vaccine in Rheumatoid Arthritis Patients
Source: J Immunol Res. 2022 Jul 28;2022:7561661. doi: 10.1155/2022/7561661 (PMC9352482; doi:10.1155/2022/7561661)
Supplement: Supplementary Materials — Figure S1: gating strategy used to identify lymphocytes, basophils, neutrophils, eosinophils, and monocytes. Figure S2: proportions of individuals with serotype-specific pneumococcal ARR ≥ 2 in healthy controls, RA 0DMARD, and RA MTX groups, after immunization with PCV13. Figure S3: serotype-specific pneumococcal antibody response in healthy controls, RA 0DMARD, and RA MTX groups, after immunization with PCV13. Table S1: distribution of antibody titers pre- and postvaccination of PCV13 in healthy controls, RA 0DMARD, and RA MTX groups. Table S2: frequencies of circulating leukocytes in RA patients, prior to contingent MTX treatment, and HC, before vaccination with PCV13. Table S3: frequencies of circulating leukocytes in RA patients with MTX treatment, before and after vaccination with PCV13. Table S4: frequencies of circulating leukocytes in RA patients without DMARD, before and after vaccination with PCV13. Table S5: frequencies of circulating leukocytes in healthy controls, before and after vaccination with PCV13. Table S6: demographic profile and characteristics of RA patients with MTX treatment, sorted in responders and nonresponders to PCV13. Table S7: frequencies of circulating leukocytes in RA patients with MTX treatment, after vaccination with PCV13, sorted in responders and nonresponders to PCV13. Table S8: expression of activation markers on circulating monocytes in MTX-treated RA patients sorted in responders and nonresponders to PCV13, pre-MTX, pre- and postvaccination. [file 7561661.f1.pdf]

Supplementary Materials

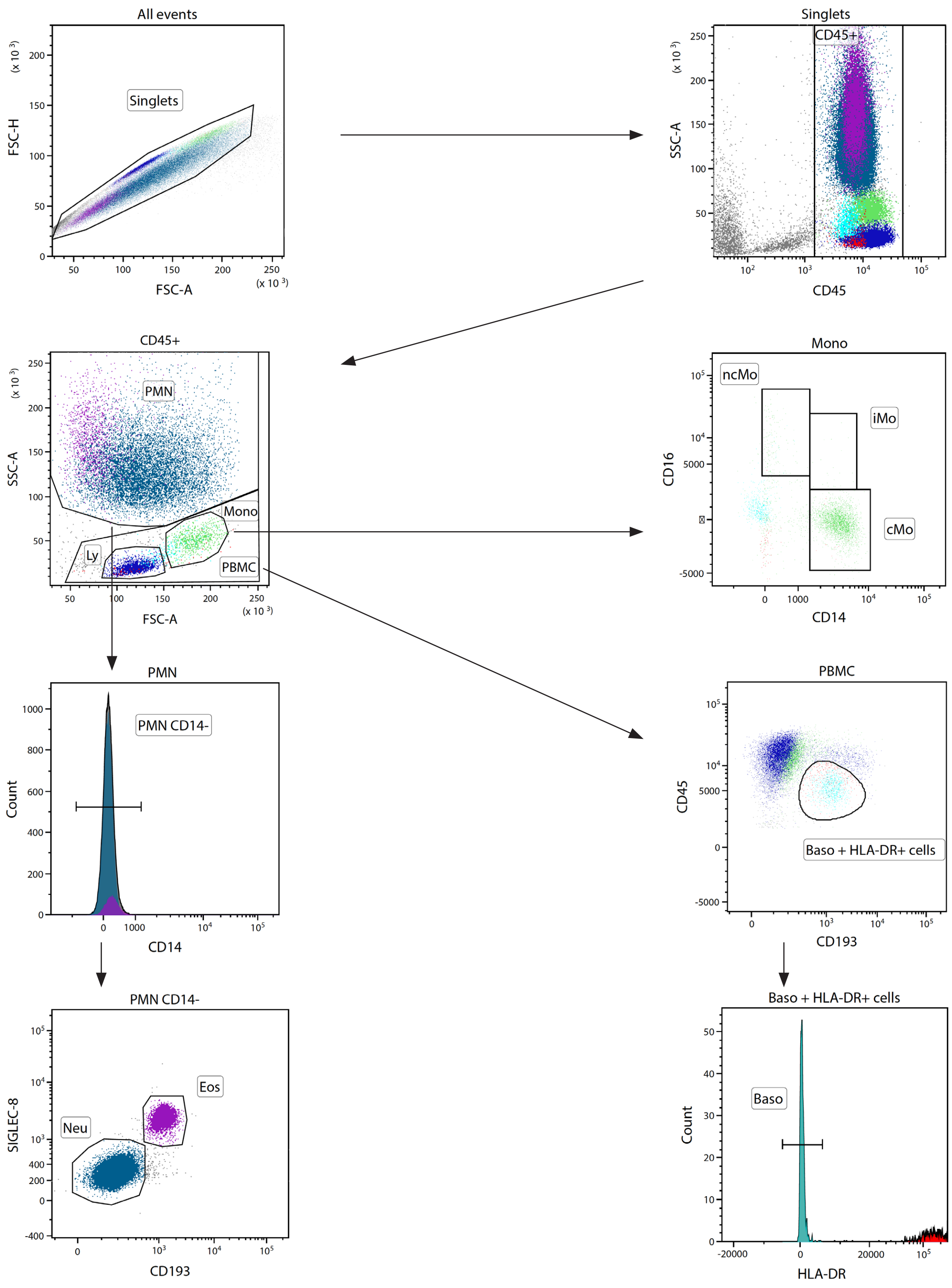

FIGURE S1. Gating strategy for lymphocytes, basophils, neutrophils, eosinophils and monocytes. A gate was first set on all events using the FSC-H/FSC-A plot for doublet discrimination. Then, CD45$^+$ single cells were gated using the SSC-A/CD45 plot. Within the CD45$^+$ single cells, peripheral blood mononuclear cells (PBMCs), granulocytes (polymorphonuclear leukocytes, PMNs), monocytes and lymphocytes were gated using the SSC-A/FSC-A plot. Monocytes were divided into classical (CD14^{++}CD16$^-$), intermediate (CD14^{++}CD16$^+$), and non-classical (CD14$^+$CD16^{++}) monocytes using the CD16/CD14 plot. Within the PMNs, CD14$^-$ cells were selected in the count/CD14 plot. Within the CD14$^-$ PMNs, neutrophils and eosinophils were gated using the Siglec-8/CD193 plot. Within the PBMCs, basophils and HLA-DR$^+$ cells were gated using the CD45/CD193 plot. Basophils were separated from plasmacytoid dendritic cells (pDCs) and other HLA-DR$^+$ cells in the count/HLA-DR plot. The figure shows an example from a rheumatoid arthritis patient with methotrexate treatment.

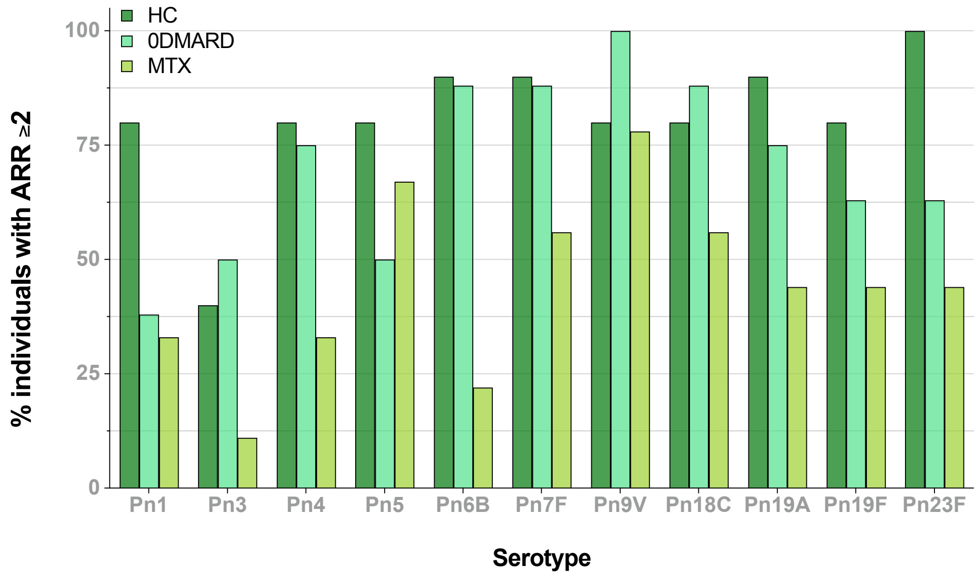

FIGURE S2. Proportions of individuals with serotype-specific pneumococcal ARR ≥2 in healthy controls, RA 0DMARD, and RA MTX groups, after immunization with PCV13. *ARR* antibody response ratio (i.e. the ratio of post- to pre-vaccination antibody levels), *RA* rheumatoid arthritis, *0DMARD* without disease-modifying antirheumatic drug treatment, *MTX* methotrexate, *PCV13* 13-valent pneumococcal conjugate vaccine, *HC* healthy control. Antibody titers were measured in 10 HC, 8 0DMARD and 9 MTX patients.

A. Antibody response ratio

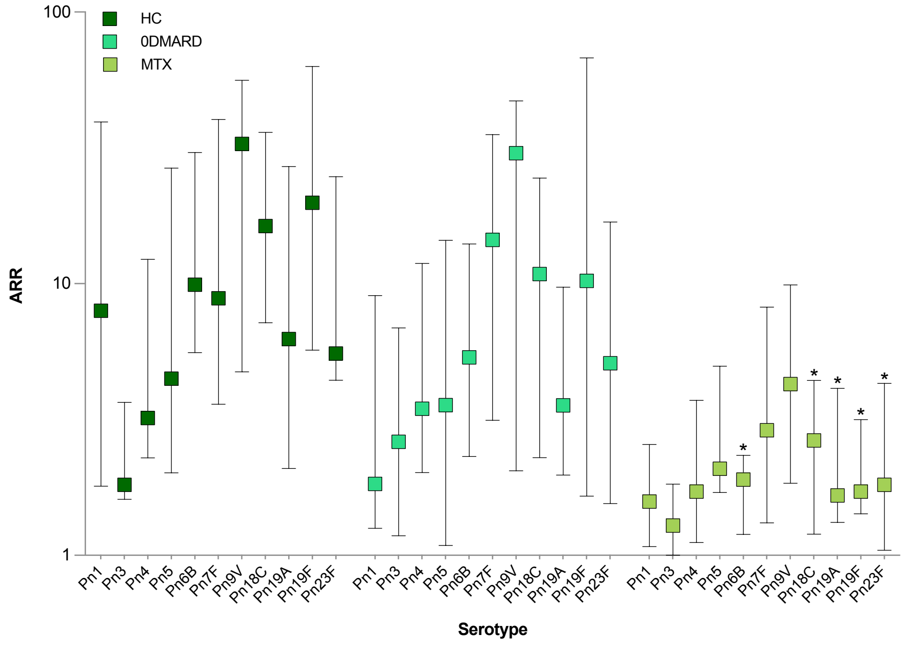

B. Pneumococcal serotype-specific IgG concentration change pre- to post-vaccination

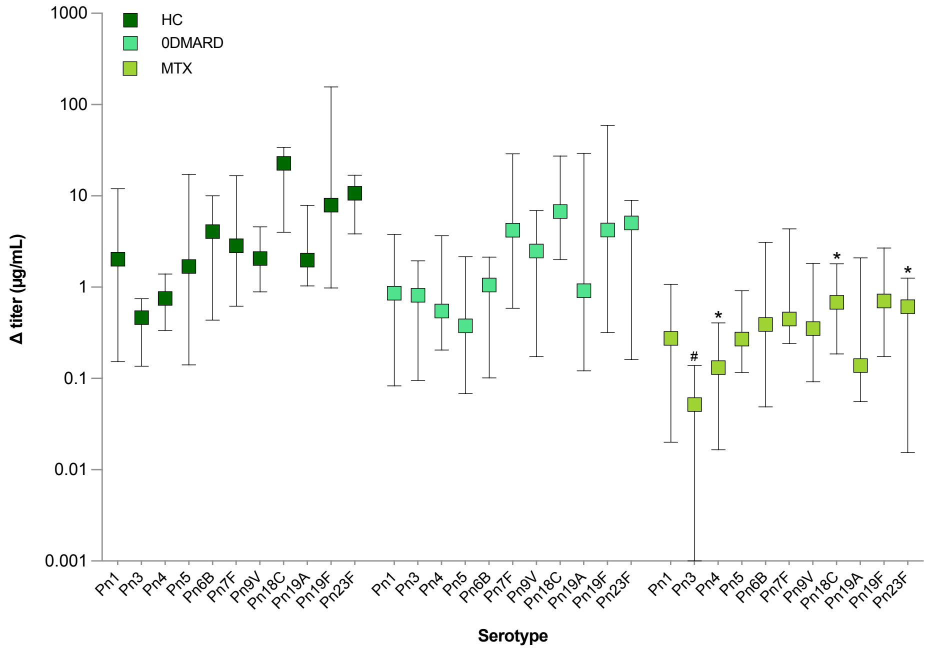

FIGURE S3. Serotype-specific pneumococcal antibody response in HC, RA 0DMARD, and RA MTX groups, after immunization with PCV13. A) Antibody response ratio, B) change in pneumococcal serotype-specific IgG concentrations (μg/mL) pre- to post-vaccination, for 11 serotypes included in PCV13. Kruskal-Wallis with Dunn's multiple comparisons test was used to calculate level of significance. Data are presented with medians and interquartile ranges. *ARR* antibody response ratio (i.e. the ratio of post- to pre-vaccination antibody levels), *HC* healthy control, *RA* rheumatoid arthritis, *0DMARD* without disease-modifying antirheumatic drug treatment, *MTX* methotrexate, *PCV13* 13-valent pneumococcal conjugate vaccine. Antibody titers were measured in 10 HC, 8 0DMARD and 9 MTX patients. * *p* <0.05 MTX vs. HC; # *p* <0.05 MTX vs. 0DMARD.

TABLE S1. Antibody titers pre- and post-vaccination of PCV13 in HC, RA 0DMARD, and RA MTX groups

	**HC**			**0DMARD**			**MTX**		
**Serotype**	Pre	Post	ARR	Pre	Post	ARR	Pre	Post	ARR
Pn1	0.33 (0.17-1.0)	5.0 (0.57-12)	8.0 (1.8-39)	0.33 (0.20-0.54)	1.2 (0.40-4.7)	1.8 (1.3-9.0)	0.57 (0.18-3.3)	0.90 (0.33-4.7)	1.6 (1.1-2.6)
Pn3	0.29 (0.11-0.56)	0.85 (0.37-1.2)	1.8 (1.6-3.7)	0.42 (0.31-2.2)	1.4 (0.78-4.9)	2.6 (1.2-6.9)	0.097 (0.029-0.85)	0.32 (0.14-0.67)	1.3 (0.94-1.8)
Pn4	0.18 (0.047-1.2)	1.2 (0.46-2.4)	3.2 (2.3-12)	0.28 (0.098-0.53)	1.0 (0.29-4.1)	3.5 (2.0-12)	0.33 (0.057-0.54)	0.53 (0.22-0.96)	1.7 (1.1-3.7)
Pn5	0.42 (0.12-0.81)	2.2 (0.48-18)	4.5 (2.0-27)	0.37 (0.075-1.6)	1.2 (0.51-2.4)	3.6 (1.1-14)	0.13 (0.063-1.5)	0.43 (0.22-3.2)	2.1 (1.7-5.0)
Pn6B	0.22 (0.13-0.68)	4.3 (0.83-10)	9.9 (5.6-30)	0.17 (0.042-0.31)	1.3 (0.13-3.7)	5.4 (2.3-14)	0.67 (0.23-2.7)	2.0 (0.27-7.5)	1.9 (1.2-2.3)
Pn7F	0.17 (0.1-0.89)	4.3 (0.73-17)	8.8 (3.6-40)	0.72 (0.16-1.8)	5.2 (0.87-31)	15 (3.1-35)	0.62 (0.12-5.1)	2.1 (0.73-9.6)	2.9 (1.3-8.2)
Pn9V	0.13 (0.020-1.0)	3.5 (1.7-6.2)	33 (4.7-56)	0.16 (0.016-0.33)	2.6 (0.23-13)	30 (2.0-47)	0.097 (0.029-0.85)	0.51 (0.21-2.7)	4.3 (1.8-9.9)
Pn18C	0.97 (0.58-2.1)	25 (5.5-35)	16 (7.2-36)	1.2 (0.41-2.0)	8.7 (3.2-29)	11 (2.3-24)	0.78 (0.32-2.8)	1.7 (1.0-6.3)	2.6 (1.2-4.4)
Pn19A	0.28 (0.14-0.86)	2.1 (1.3-15)	6.3 (2.1-27)	0.95 (0.087-3.3)	2.7 (0.24-31)	3.6 (2.0-9.7)	0.69 (0.095-2.9)	1.6 (0.20-3.5)	1.7 (1.3-4.1)
Pn19F	1.1 (0.29-1.8)	8.9 (1.8-159)	20 (5.7-63)	0.51 (0.054-5.3)	13 (0.35-63)	10 (1.7-68)	1.7 (0.22-5.3)	3.5 (0.66-6.7)	1.7 (1.4-3.2)
Pn23F	0.92 (0.44-3.3)	13 (5.8-21)	5.5 (4.4-25)	0.77 (0.37-1.9)	7.1 (1.1-11)	5.1 (1.5-17)	0.59 (0.32-1.2)	1.6 (0.56-3.2)	1.8 (1.0-4.3)

Pneumococcal serotype-specific IgG concentrations (μg/mL) for 11 serotypes included in PCV13. Data are presented with medians and interquartile ranges. *PCV13* 13-valent pneumococcal conjugate vaccine, *HC* healthy control, *RA* rheumatoid arthritis, *0DMARD* without disease-modifying antirheumatic drug treatment, *MTX* methotrexate, *ARR* antibody response ratio (i.e. the ratio of post- to pre-vaccination antibody levels). Pneumococcal serotype-specific IgG concentrations were measured in 10 HC, 8 0DMARD and 9 MTX patients. Samples with concentrations below the lower limit of detection (for serotype 9V, n=2, one 0DMARD patient and one HC, both pre-vaccination) were set to 0.

TABLE S2. Frequencies of circulating leukocytes in RA patients, prior to contingent MTX treatment, and HC, before vaccination with PCV13

**Phenotype**	**Healthy controls** (n=13[a])	**RA patients** (n=22[b])	***p* value**
Monocytes (% of leukocytes)	5.62 (4.82-7.58)	6.04 (5.19-7.52)	ns
Monocytes (10^9/L)	0.304 (0.241-0.440)	0.430 (0.308-0.730)	ns
$CD14^{++}CD16^{-}$ (% of monocytes)	84.5 (79.3-87.8)	86.4 (83.6-90.6)	ns
$CD14^{++}CD16^{-}$ (10^9/L)	0.266 (0.190-0.377)	0.407 (0.272-0.684)	ns
$CD14^{++}CD16^{+}$ (% of monocytes)	2.62 (2.04-4.14)	4.31 (1.90-5.10)	ns
$CD14^{++}CD16^{+}$ (10^9/L)	0.00632 (0.00440-0.00745)	0.0146 (0.0101-0.0250)	0.03
$CD14^{+}CD16^{++}$ (% of monocytes)	13.3 (9.11-16.0)	8.89 (5.66-12.2)	0.03
$CD14^{+}CD16^{++}$ (10^9/L)	0.0398 (0.0308-0.0669)	0.0518 (0.0302-0.0552)	ns
Granulocytes (% of leukocytes)	60.1 (43.5-69.6)	66.3 (58.0-81.4)	ns
Granulocytes (10^9/L)	3.39 (2.57-4.76)	5.25 (3.27-10.4)	ns
Basophils (% of leukocytes)	0.940 (0.570-1.24)	0.915 (0.628-1.15)	ns
Basophils (10^9/L)	0.0567 (0.0305-0.0605)	0.0635 (0.0372-0.121)	ns
Eosinophils (% of leukocytes)	3.00 (1.60-3.29)	2.40 (1.12-4.66)	ns
Eosinophils (10^9/L)	0.0746 (0.0525-0.146)	0.140 (0.0769-0.305)	ns
Neutrophils (% of leukocytes)	57.9 (39.2-66.1)	60.5 (55.0-77.9)	ns
Neutrophils (10^9/L)	3.14 (2.45-4.65)	4.91 (3.17-10.2)	ns
Lymphocytes (% of leukocytes)	24.8 (20.9-42.0)	19.2 (9.74-31.4)	ns
Lymphocytes (10^9/L)	1.04 (0.817-1.29)	1.11 (0.730-1.48)	ns

Frequencies of circulating monocytes, granulocytes and lymphocytes analyzed in patients with RA (prior to contingent MTX treatment) and healthy controls, before administration of PCV13, using flow cytometry. Mann-Whitney *U* test was used to calculate level of significance. Data are presented with medians and interquartile ranges. *RA* rheumatoid arthritis, *MTX* methotrexate, *HC* healthy control, *PCV13* 13-valent pneumococcal conjugate vaccine, *ns* not significant. [a]n equals 5 for absolute values, [b]n equals 15 for absolute values.

TABLE S3. Frequencies of circulating leukocytes in RA patients with MTX treatment, before and after vaccination with PCV13

**Phenotype**	**MTX Pre-PCV13** (n=11[a])	**MTX Post-PCV13** (n=11[a])	***p* value**
Monocytes (% of leukocytes)	6.38 (4.83-11.2)	7.86 (3.42-10.9)	ns
Monocytes (10^9/L)	0.428 (0.296-0.505)	0.309 (0.120-0.656)	ns
$CD14^{++}CD16^{-}$ (% of monocytes)	83.2 (81.0-89.5)	86.5 (80.1-90.1)	ns
$CD14^{++}CD16^{-}$ (10^9/L)	0.356 (0.164-0.439)	0.248 (0.0838-0.541)	ns
$CD14^{++}CD16^{+}$ (% of monocytes)	3.35 (1.66-7.13)	3.26 (1.39-4.99)	ns
$CD14^{++}CD16^{+}$ (10^9/L)	0.0169 (0.00818-0.0498)	0.00861 (0.00430-0.0231)	ns
$CD14^{+}CD16^{++}$ (% of monocytes)	9.68 (7.05-16.1)	10.4 (6.63-13.9)	ns
$CD14^{+}CD16^{++}$ (10^9/L)	0.0507 (0.0409-0.0578)	0.0445 (0.0414-0.0572)	ns
Granulocytes (% of leukocytes)	65.1 (60.0-77.1)	71.0 (60.3-81.0)	ns
Granulocytes (10^9/L)	3.74 (2.48-5.87)	3.86 (2.91-4.95)	ns
Basophils (% of leukocytes)	1.21 (1.03-1.37)	1.17 (1.11-1.49)	ns
Basophils (10^9/L)	0.0662 (0.0550-0.111)	0.0620 (0.0499-0.0974)	ns
Eosinophils (% of leukocytes)	3.57 (2.54-5.84)	5.73 (2.32-8.17)	ns
Eosinophils (10^9/L)	0.162 (0.137-0.459)	0.246 (0.109-0.310)	ns
Neutrophils (% of leukocytes)	59.9 (56.4-68.5)	61.7 (55.8-73.5)	ns
Neutrophils (10^9/L)	3.43 (2.29-5.27)	3.69 (2.76-4.54)	ns
Lymphocytes (% of leukocytes)	21.4 (12.3-27.4)	15.5 (9.77-29.0)	ns
Lymphocytes (10^9/L)	1.11 (0.693-1.55)	0.663 (0.638-1.29)	0.02

Frequencies of circulating monocytes, granulocytes and lymphocytes analyzed in RA patients with MTX treatment for 6-12 weeks, before and 6-7 days after administration of PCV13, using flow cytometry. Wilcoxon matched-pairs signed rank test was used to calculate level of significance. Data are presented with medians and interquartile ranges. *RA* rheumatoid arthritis, *MTX* methotrexate, *PCV13* 13-valent pneumococcal conjugate vaccine, *ns* not significant. [a]n equals 7 for absolute values.

TABLE S4. Frequencies of circulating leukocytes in RA patients without DMARD, before and after vaccination with PCV13

**Phenotype**	**0DMARD Pre-PCV13** (n=10[a])	**0DMARD Post-PCV13** (n=10[a])	***p* value**
Monocytes (% of leukocytes)	5.84 (4.90-7.65)	7.18 (5.44-8.55)	ns
Monocytes (10^9/L)	0.543 (0.419-0.854)	0.454 (0.393-0.725)	ns
$CD14^{++}CD16^{-}$ (% of monocytes)	87.9 (82.8-91.5)	88.1 (84.7-91.5)	ns
$CD14^{++}CD16^{-}$ (10^9/L)	0.464 (0.332-0.775)	0.450 (0.213-0.659)	ns
$CD14^{++}CD16^{+}$ (% of monocytes)	4.77 (2.21-5.45)	2.56 (1.02-4.77)	ns
$CD14^{++}CD16^{+}$ (10^9/L)	0.0218 (0.0101-0.0250)	0.00783 (0.00667-0.0105)	ns
$CD14^{+}CD16^{++}$ (% of monocytes)	7.85 (4.68-11.6)	8.34 (5.80-11.5)	ns
$CD14^{+}CD16^{++}$ (10^9/L)	0.0545 (0.0428-0.0614)	0.0468 (0.0311-0.0865)	ns
Granulocytes (% of leukocytes)	66.0 (58.0-82.7)	69.0 (52.9-78.7)	ns
Granulocytes (10^9/L)	7.64 (2.91-10.5)	4.58 (3.29-8.10)	ns
Basophils (% of leukocytes)	0.895 (0.548-1.13)	1.07 (0.878-1.51)	0.002
Basophils (10^9/L)	0.121 (0.0372-0.135)	0.123 (0.0449-0.131)	ns
Eosinophils (% of leukocytes)	1.71 (1.03-3.66)	1.88 (1.58-3.65)	ns
Eosinophils (10^9/L)	0.0984 (0.0376-0.408)	0.148 (0.0817-0.197)	ns
Neutrophils (% of leukocytes)	59.4 (56.3-80.2)	64.2 (45.7-75.6)	ns
Neutrophils (10^9/L)	7.52 (2.77-10.2)	4.40 (2.42-7.77)	ns
Lymphocytes (% of leukocytes)	20.1 (8.85-31.4)	16.9 (11.0-31.4)	ns
Lymphocytes (10^9/L)	1.11 (0.980-1.36)	1.18 (0.917-1.74)	ns

Frequencies of circulating monocytes, granulocytes and lymphocytes analyzed in RA patients without DMARD, before and 6-7 days after administration of PCV13, using flow cytometry. Wilcoxon matched-pairs signed rank test was used to calculate level of significance. Data are presented with medians and interquartile ranges. *RA* rheumatoid arthritis, *DMARD* disease-modifying antirheumatic drug treatment, *PCV13* 13-valent pneumococcal conjugate vaccine, *ns* not significant. [a]n equals 7 for absolute values.

TABLE S5. Frequencies of circulating leukocytes in healthy controls, before and after vaccination with PCV13

**Phenotype**	**HC**<br>**Pre-PCV13**<br>(n=12[a])	**HC**<br>**Post-PCV13**<br>(n=12[a])	***p* value**
Monocytes (% of leukocytes)	5.59 (4.76-7.61)	6.21 (4.59-8.26)	ns
Monocytes (10^9/L)	0.301 (0.212-0.503)	0.327 (0.234-0.360)	ns
$CD14^{++}CD16^{-}$ (% of monocytes)	84.0 (79.1-88.1)	82.9 (78.3-86.2)	ns
$CD14^{++}CD16^{-}$ (10^9/L)	0.263 (0.158-0.429)	0.276 (0.180-0.290)	ns
$CD14^{++}CD16^{+}$ (% of monocytes)	2.83 (2.09-4.34)	2.93 (2.34-3.42)	ns
$CD14^{++}CD16^{+}$ (10^9/L)	0.00585 (0.00423-0.00771)	0.00708 (0.00559-0.00897)	ns
$CD14^{+}CD16^{++}$ (% of monocytes)	13.8 (9.10-16.8)	14.8 (10.8-18.9)	ns
$CD14^{+}CD16^{++}$ (10^9/L)	0.0460 (0.0275-0.0741)	0.0489 (0.0383-0.0655)	ns
Granulocytes (% of leukocytes)	59.4 (41.8-70.3)	58.2 (46.0-73.3)	ns
Granulocytes (10^9/L)	3.90 (2.25-4.86)	2.22 (1.91-3.26)	ns
Basophils (% of leukocytes)	0.930 (0.550-1.20)	0.870 (0.595-1.26)	ns
Basophils (10^9/L)	0.0450 (0.0291-0.0581)	0.0449 (0.0233-0.0742)	ns
Eosinophils (% of leukocytes)	2.74 (1.46-3.10)	2.95 (1.91-4.68)	0.01
Eosinophils (10^9/L)	0.0720 (0.0440-0.103)	0.0932 (0.0547-0.112)	ns
Neutrophils (% of leukocytes)	56.2 (37.4-67.0)	54.0 (41.3-69.5)	ns
Neutrophils (10^9/L)	3.78 (2.14-4.74)	2.10 (1.79-3.10)	ns
Lymphocytes (% of leukocytes)	27.5 (20.3-42.2)	30.9 (15.9-37.6)	ns
Lymphocytes (10^9/L)	1.02 (0.727-1.32)	1.55 (0.922-2.03)	ns

Frequencies of circulating monocytes, granulocytes and lymphocytes analyzed in healthy controls, before and 6-7 days after administration of PCV13, using flow cytometry. Wilcoxon matched-pairs signed rank test was used to calculate level of significance. Data are presented with medians and interquartile ranges. *PCV13* 13-valent pneumococcal conjugate vaccine, *HC* healthy controls, *ns* not significant. [a]n equals 4 for absolute values.

TABLE S6. Demographic profile and characteristics of RA patients with MTX treatment, sorted in responders and non-responders to PCV13

	**Responders** (n=5)	**Non-responders** (n=4)	***p* value**
Age, years, median (IQR)	59.8 (58.9-64.3)	68.9 (55.7-79.2)	ns
Female/male, n (%)	4/1 (80/20)	4/0 (100/0)	
Disease duration, years, median (IQR)	0.3 (0.2-1.4)	0.4 (0.2-21.6)	ns
DAS28 at MTX start, median (IQR)[a]	5.1 (4.8-7.0)	5.6 (5.1-6.4)	ns
DAS28 at vaccination, median (IQR)[a]	3.6 (2.9-5.0)	4.7 (3.2-5.9)	ns
CRP at MTX start, mg/L, median (IQR)[b]	5.1 (2.8-22.5)	31.0 (11.6-65.3)	ns
CRP at vaccination, mg/L, median (IQR)[b]	2.8 (1.6-6.8)	4.5 (1.7-10.7)	ns
ESR at MTX start, mm, median (IQR)[c]	32 (17-61)	74 (55-85)	ns
ESR at vaccination, mm, median (IQR)[c]	12 (8-25)	43 (36-59)	0.03
Prednisolone at vaccination, n (%)	3 (60)	1 (25)	
- dose in treated, mg/day, median (IQR)	2.5 (2.5-5)	15	
Methotrexate at vaccination, mg/week, median (IQR)	20 (17.5-25.0)	17.5 (15-23.8)	ns

Positive antibody response was defined as an antibody response ratio (ARR, i.e., the ratio of post- to pre-vaccination antibody levels) ≥2, in >50% of serotypes. *RA* rheumatoid arthritis, *MTX* methotrexate, *PCV13* 13-valent pneumococcal conjugate vaccine, *ns* not significant, *IQR* interquartile range, *DAS28* Disease Activity Score 28 joints examined, *CRP* (P-CRP) C-reactive protein, *ESR* (B-ESR) erythrocyte sedimentation rate. Antibody titers were available in 9 of 11 patients on MTX. [a]On a scale 0-10 DAS28 >5.1 high, 5.1-3.2 moderate, <3.2-2.6 low disease activity, <2.6 remission. [b]Reference range <3.0 mg/L. [c]Reference range <30 mm (female), <20 mm (male).

TABLE S7. Frequencies of circulating leukocytes in RA patients with MTX treatment, after vaccination with PCV13, sorted in responders and non-responders to PCV13

**Phenotype**	**MTX post-PCV13 Responders** (n=5)[a]	**MTX post-PCV13 Non-responders** (n=4)[b]	***p* value**
Monocytes (% of leukocytes)	9.35 (7.18-12.5)	2.85 (1.94-3.60)	0.02
Monocytes (10^9/L)	0.693 (0.578-0.809)	0.278 (0.107-0.294)	
$CD14^{++}CD16^{-}$ (% of monocytes)	90.1 (83.3-92.8)	82.1 (61.8-87.7)	ns
$CD14^{++}CD16^{-}$ (10^9/L)	0.620 (0.499-0.741)	0.218 (0.0916-0.260)	
$CD14^{++}CD16^{+}$ (% of monocytes)	2.40 (1.75-3.71)	5.23 (1.61-7.53)	ns
$CD14^{++}CD16^{+}$ (10^9/L)	0.0205 (0.0170-0.0240)	0.00387 (0.00274-0.0216)	
$CD14^{+}CD16^{++}$ (% of monocytes)	6.63 (4.94-13.9)	12.3 (10.6-31.2)	ns
$CD14^{+}CD16^{++}$ (10^9/L)	0.0524 (0.0506-0.0542)	0.0315 (0.0112-0.0386)	
Granulocytes (% of leukocytes)	66.8 (38.1-73.2)	78.4 (64.2-82.6)	ns
Granulocytes (10^9/L)	3.93 (2.91-4.94)	4.49 (3.81-7.15)	
Basophils (% of leukocytes)	1.21 (0.815-2.05)	1.33 (1.12-1.63)	ns
Basophils (10^9/L)	0.0570 (0.0201-0.0940)	0.0844 (0.0545-0.144)	
Eosinophils (% of leukocytes)	6.05 (2.77-8.48)	6.80 (4.00-8.50)	ns
Eosinophils (10^9/L)	0.404 (0.360-0.448)	0.269 (0.260-0.677)	
Neutrophils (% of leukocytes)	59.5 (30.4-66.9)	69.6 (58.3-74.0)	ns
Neutrophils (10^9/L)	3.47 (2.53-4.40)	4.24 (3.48-6.32)	
Lymphocytes (% of leukocytes)	18.0 (12.6-47.5)	17.3 (10.7-26.7)	ns
Lymphocytes (10^9/L)	0.867 (0.401-1.33)	0.804 (0.699-2.21)	

Frequencies of circulating monocytes, granulocytes and lymphocytes analyzed in RA patients on MTX, 6-7 days after administration of PCV13, using flow cytometry. Positive antibody response was defined as an antibody response ratio (ARR, i.e., the ratio of post- to pre-vaccination antibody levels) ≥2, in >50% of serotypes. Mann-Whitney *U* test was used to calculate level of significance. Data are presented with medians and interquartile ranges. *RA* rheumatoid arthritis, *MTX* methotrexate, *PCV13* 13-valent pneumococcal conjugate vaccine, *ns* not significant. Antibody titers were available in 9 of 11 patients on MTX. [a]n equals 2 for absolute values, [b]n equals 3 for absolute values.

TABLE S8. Expression of activation markers on circulating monocytes in MTX-treated RA patients, sorted in responders and non-responders to PCV13, pre-MTX, pre- and post-vaccination

	Pre-MTX		Pre-vaccination		Post-vaccination	
**Phenotype (MdFI)**	**Responders** (n=4)	**Non-responders** (n=4)	**Responders** (n=5)	**Non-responders** (n=4)	**Responders** (n=5)	**Non-responders** (n=4)
Monocytes						
CD11b	9602 (2612-18832)	9986 (2648-25448)	11633 (5180-16218)	6322 (2414-13661)	9181 (5578-22520)	10094 (7282-14660)
CD62L	695 (425-1581)	1054 (733-1199)	1989 (1058-3265)	2238 (952-3823)	2856 (1356-3589)	1351 (1288-1444)
CD69	8607 (3401-17278)	14576 (5223-18830)	7257 (5009-17391)	9376 (3932-11785)	6605 (5570-8101)	15275 (4248-23469)
CD80	56.5 (8.72-110)	110 (91.2-124)	92.9 (41.9-113)	86.0 (70.2-100)	82.7 (42.9-118)	103 (91.8-114)
HLA-DR	26833 (615-64262)	67996 (60697-70805)	62125 (13851-91514)	45819 (34290-62929)	46506 (27191-69181)	75898 (41110-84187)
$CD14^{++}CD16^{-}$ monocytes						
CD11b	10073 (2743-19037)	9915 (2598-26032)	11976 (5258-16541)	6300 (2577-17065)	9424 (5761-22857)	10438 (7658-14806)
CD62L	835 (684-1713)	1092 (739-1264)	2105 (1096-3848)	2150 (593-4277)	3488 (1494-4203)	1595 (1532-2388)
CD69	8747 (3418-18486)	15103 (5364-19466)	7887 (5294-17949)	12763 (4022-17240)	7230 (5763-8526)	17290 (5090-24173)
CD80	57.8 (8.72-110)	108 (91.6-126)	89.9 (41.8-115)	93.3 (71.0-121)	82.5 (43.4-117)	106 (90.5-118)
HLA-DR	25472 (463-67998)	67931 (59493-72839)	61890 (13324-90483)	56172 (36455-84264)	43711 (26284-67958)	72378 (38324-86355)
$CD14^{++}CD16^{+}$ monocytes						
CD11b	14396 (3239-24934)	21242 (12228-36554)	14761 (10456-21963)	13422 (9658-19581)	13851 (7169-26477)	12755 (8622-17535)
CD62L	270 (0.00-1094)	632 (483-1606)	609 (212-1162)	779 (340-1314)	821 (0.00-1059)	775 (677-907)
CD69	11273 (6468-18538)	15333 (6096-17293)	6964 (4865-18160)	11111 (4939-17253)	7091 (4549-10069)	17405 (5404-26279)
CD80	39.4 (0.390-116)	130 (76.5-168)	86.1 (30.7-99.3)	65.6 (58.5-102)	68.4 (12.9-125)	113 (95.4-127)
HLA-DR	123479 (6497-248338)	255575 (243622-257377)	254348 (76265-255919)	250711 (159385-258177)	216815 (119694-257849)	196334 (125843-256588)
$CD14^{+}CD16^{++}$ monocytes						
CD11b	3992 (1275-7990)	6614 (2148-9701)	3833 (1528-10643)	3724 (1541-8960)	5532 (1451-8247)	7985 (2480-12067)
CD62L	264 (0.00-723)	789 (501-1559)	493 (293-1155)	1478 (329-4196)	235 (57.2-402)	438 (197-1068)
CD69	2359 (1325-4425)	2047 (743-3545)	987 (502-2332)	1250 (573-1586)	1112 (415-1536)	2570 (1248-8524)
CD80	53.3 (15.6-122)	95.9 (87.2-135)	89.4 (45.0-108)	74.5 (68.8-89.6)	94.3 (43.1-139)	87.8 (76.0-113)
HLA-DR	4478 (546-46090)	1392 (883-61580)	24409 (2087-65829)	24493 (2073-65583)	48768 (8670-77680)	64207 (22708-144056)

Levels of surface expression of CD11b, CD62L, CD69, CD80 and HLA-DR on circulating monocytes, analyzed in patients with RA (responders and non-responders to PCV13), pre-MTX treatment, pre- and post-vaccination, using flow cytometry, as a measurement of activation. There were no statistical differences in expression of activation markers on monocytes and subsets between responders and non-responders at the three different time points. Mann-Whitney *U* test was used to calculate level of significance. Data are presented with medians and interquartile ranges. *MTX* methotrexate, *RA* rheumatoid arthritis, *PCV13* 13-valent pneumococcal conjugate vaccine, *MdFI* median fluorescence intensity.
